# Advances in the interplay between mechanical forces and inflammatory immunity in keloid formation

**DOI:** 10.3389/fimmu.2026.1792028

**Published:** 2026-04-24

**Authors:** Simin Zhou, Lei Wang, Nannan Chen, Jinqiao Liu, Rongjia Zhang, Shiqi Zhou, Yiling Liu, Wenyan Zhu, Xiaodong Chen, Run Meng

**Affiliations:** 1 Department of Immunology, Medical School, Nantong University, Nantong, China; 2 Department of Dermatology, Affiliated Hospital of Nantong University, Nantong, China

**Keywords:** fibrosis, inflammatory response, keloid, mechanical stress, signaling pathway, therapeutic targets

## Abstract

Keloids are chronic inflammatory fibroproliferative disorders characterized by invasive growth beyond the original wound margins and a high recurrence rate, substantially impairing patients’ quality of life. Their pathogenesis is complex and arises from the synergistic interplay among the mechanical microenvironment, chronic inflammation, and profibrotic signaling networks. The inflammatory and proliferative phases of normal wound healing are pathologically prolonged, with sustained activation of fibroblasts and myofibroblasts, resulting in excessive extracellular matrix (ECM) deposition and aberrant remodeling. Within the immune microenvironment, infiltrating M2 macrophages and Th2/Th17 cells, among others, secrete a broad array of cytokines, thereby establishing a chronic inflammatory circuit. Mechanical forces act as a pivotal driving factor: stress concentration in high-tension regions and activation of fibroblast mechanotransduction pathways (e.g., Hippo–YAP/TAZ and integrin–FAK) interact with inflammatory responses to form a self-amplifying “mechanical force–inflammation–fibrosis” positive feedback loop, exacerbating disease progression. Current management primarily relies on multimodal regimens such as surgery combined with radiotherapy and intralesional pharmacologic injections, yet remains challenged by high recurrence rates and marked heterogeneity. In recent years, tension-reduction approaches grounded in mechanomodulation and targeted therapies against inflammatory pathways such as JAK/STAT and IL-4/IL-13 have shown progress, and integrated “mechanics–inflammation” combinatorial interventions are emerging as a new direction. The review summarizes the pathophysiological features of keloids and elucidates the roles and crosstalk of mechanical forces and inflammation, thereby providing a novel theoretical framework centered on mechano-immunology for future individualized clinical management of keloids.

## 1 Pathophysiological basis and clinical manifestations of keloids

### 1.1 Comparison between normal wound healing and pathological scarring

Keloids are a class of chronic fibroproliferative skin lesions that often involve the dermis and subcutaneous tissue. Their most clinically important characteristic is the persistent growth of the lesion beyond the boundaries of the original injury, with infiltrative extension into the surrounding normal skin and little tendency toward spontaneous regression (1). The high recurrence rate even after interventions such as surgical excision suggests that keloids are not merely a form of excessive scarring, but rather represent an abnormal fibroproliferative state that is difficult to terminate (2). Keloids may be triggered by trauma, burns, surgical incisions, acne, and other insults; however, in some cases the preceding injury is minimal or even unrecognizable, indicating that genetic susceptibility, individual variation, and dysregulation of immune-inflammatory and fibrotic control play important roles in their pathogenesis (3).

Unlike normal scars, which are usually confined to the area of injury and gradually soften and stabilize over time, keloids are more likely to remain in a highly active state. More pronounced elevation, erythema, and worsening symptoms can often be observed at the lesion margins, suggesting an underlying biological basis for their continued progression (4). Recent studies have proposed that keloids more closely resemble a localized fibroproliferative disorder with tumor-like features (5). Their tumor-like nature is mainly reflected in persistent local proliferation and invasive expansion, sustained angiogenesis and immune cell infiltration, and long-term imbalance in matrix remodeling. Unlike malignant tumors, keloids usually do not undergo distant metastasis, but their long-term local progression and high recurrence share many similarities with a tumor-like microenvironment driven by chronic inflammation (6). Notably, the tumor-like biological behaviors exhibited by solid tumors are usually closely associated with alterations in their mechanical microenvironment. Increased matrix stiffness, enhanced collagen crosslinking, and elevated solid stress not only promote tumor cell migration and invasion, but also alter the pattern of immune cell infiltration by affecting vascular perfusion and creating physical barriers, ultimately facilitating the persistence and amplification of inflammation. Keloids likewise exhibit similar features, including local matrix stiffening and concentrated mechanical tension, suggesting that their tumor-like pathological microenvironment may also involve interactions among mechanical forces, immunity, and inflammation (7–9). This provides a pathobiological basis for the subsequently proposed positive feedback model of “mechanical force–inflammation–fibrosis.”

### 1.2 Histological and cytological characteristics and mechanistic overview

The histopathological features of keloids are characterized by disorganized collagen architecture, an imbalance in the ratio of type I to type III collagen, and a reduction or absence of elastic fibers, suggesting a long-term disequilibrium between matrix production and degradation that is associated with abnormal mechanical properties. At the cellular level, persistent activation of fibroblasts and myofibroblasts is central, manifested by sustained high expression of α-SMA and multiple extracellular matrix (ECM) components, enhanced proliferation, and suppressed apoptosis (10). Single-cell studies have further revealed marked cellular heterogeneity, including enrichment of fibroblast subpopulations associated with high collagen expression and mechanical stimulation, and have suggested that populations such as endothelial cells and keratinocytes may also participate in the infiltrative behavior of the lesion (11). With respect to the immune microenvironment, various immune cells, including M2 macrophages, mast cells, and Th2/Th17 cells, are commonly observed within lesions, where they maintain a profibrotic state through cytokine secretion and intercellular interactions. At the molecular level, the disease involves coordinated dysregulation of multiple signaling pathways, with commonly reported aberrantly activated axes including TGF-β/Smad, Hippo-YAP/TAZ, integrin-FAK, JAK/STAT, and NF-κB (12, 13). When matrix stiffening and cytoskeletal tension increase, YAP/TAZ undergo nuclear translocation. In addition to regulating transcriptional programs related to ECM synthesis and cell proliferation, studies suggest that YAP/TAZ can also interact with inflammation-related transcriptional networks such as NF-κB, upregulating the expression of cytokines and chemokines including IL-6 and IL-8, thereby directly converting matrix stiffening and mechanical tension signals into key signals that drive immune cell recruitment and the persistence of chronic inflammation (14, 15). In addition, familial aggregation and population differences suggest that keloids may also have genetic and epigenetic susceptibility; for example, genome-wide association studies (GWAS) have identified multiple susceptibility loci in East Asian populations, among which the candidate gene NEDD4 at the 15q21.3 locus may be associated with inflammatory responses and fibrotic progression in keloids (16, 17).

### 1.3 Clinical manifestations and disease burden

Keloids are more commonly observed in adolescents and young adults, and some studies suggest a slight female predominance (18). Lesions commonly occur in areas subjected to relatively high mechanical tension, such as the presternal region, shoulders, upper arms, earlobes, and mandible, and may become markedly elevated with crab-claw-like infiltrative extension (19). Patients often experience pruritus, burning pain, or stinging sensations, and these symptoms may worsen at night, after exercise, or during periods of increased psychological stress, thereby affecting sleep and daily functioning. When lesions are located near joints or in special regions such as the neck, they may lead to restricted movement and pain or discomfort in the affected area. In addition to physical symptoms, keloids impose a substantial psychological burden because of their appearance and chronic symptoms, particularly when lesions are located on exposed body sites, where they are more likely to cause emotional distress and impaired social functioning. Sleep disturbances caused by long-term pruritus and pain may further aggravate psychological stress (20). In addition, the risk of keloid development varies markedly across populations worldwide, with individuals of darker skin tones and certain African and Asian populations exhibiting a relatively higher risk, suggesting that the disease burden is not uniformly distributed across regions and populations and may also impose a substantial symptom burden and impair quality of life (21, 22). The following sections will focus on the mechanical basis of keloid initiation and progression, the underlying inflammatory and immune mechanisms, and the interplay between these two processes.

## 2 Mechanisms of keloid formation

A growing body of evidence indicates that keloid formation is not driven by abnormalities in a single cell type or signaling pathway, but rather results from the combined effects of multiple cellular components, alterations in the local microenvironment, and interconnected signaling networks. In recent years, the application of technologies such as single-cell sequencing and spatial transcriptomics has further revealed the complexity of keloids at the levels of cellular composition, microenvironmental features, and molecular regulation, and has provided new evidence for understanding their initiation and progression within an integrated framework of “cells–microenvironment–signaling pathways.”

### 2.1 Fibroblasts/myofibroblasts and extracellular matrix remodeling

The excessive proliferation, apoptosis resistance, and continuous ECM production of fibroblasts—the principal effector cells in keloid formation—directly drive the progressive fibrotic expansion of keloids (23). During normal wound healing, fibroblasts transiently differentiate into α-SMA^+^ myofibroblasts in response to cues such as TGF-β and mechanical tension, contributing to wound-edge contraction and ECM deposition (24). In keloids, however, α-SMA^+^ myofibroblasts are markedly increased and persist over time, remaining chronically activated and characterized by abundant stress fibers and high contractility, thereby producing excessive ECM and serving as the major cellular basis for lesion thickening and tissue stiffening (25).

Keloids harbor fibroblasts and myofibroblasts with strong proliferative capacity and elevated apoptotic thresholds, which are more sensitive to TGF-β and mechanical stimuli, and markedly promote the production of ECM components such as type I/III collagen and fibronectin, while showing deficient expression of degradative enzymes (e.g., MMPs) or suppression by their inhibitors, TIMPs (26). From the perspective of the ECM, disorganized collagen architecture, excessive cross-linking, paucity of elastic fibers, and an abnormal type I/type III collagen ratio within connective tissue can all increase tissue stiffness.

A stiff and dense ECM increases cytoskeletal tension via the integrin–focal adhesion complex–FAK and RhoA/ROCK signaling pathways, promoting the nuclear translocation of YAP/TAZ and activation of profibrotic genes, thereby further reinforcing fibroblast and myofibroblast proliferation, contractility, and ECM synthesis (27). Prolonged, sustained contraction of myofibroblasts continuously compresses and reorganizes collagen fibers, progressively stiffening the local ECM. As matrix stiffness increases, myofibroblasts are more likely to maintain an activated state and further enhance contractile and synthetic activity, promoting additional ECM deposition and crosslinking, which in turn exacerbates matrix stiffening. This process provides an important cellular and molecular basis for the increased tissue density, elevated stiffness, and limited reversibility of keloids.

### 2.2 Immune microenvironment and chronic inflammation

Keloids are not only fibroproliferative disorders but also chronic inflammatory diseases. Both immune-cell populations and cytokine networks are markedly remodeled in keloids, with a notable increase in innate immune cells such as M2 macrophages, mast cells, and dendritic cells, among which M2 macrophages predominate. By secreting profibrotic mediators including TGF-β, IL-10, and VEGF, M2 macrophages induce fibroblast activation and ECM production, and also participate in angiogenesis and local immune responses (28, 29).

In terms of adaptive immunity, the proportions of Th2 and Th17 cells are increased within keloid lesions, and Th2/Th17 cells can interact with fibroblasts to form the canonical pro-fibrotic “Th2–M2–fibroblast” loop. Regulatory T cells (Tregs) can promote fibrosis while suppressing local inflammation through the release of IL-10 and TGF-β1 (30).

Lee et al. (2022) (31) reported that IL-17 suppresses autophagy in keloids and modulates inflammatory cell apoptosis and fibrogenesis via STAT3 and HIF-1α. These findings further highlight the impact of IL-17 on the immune microenvironment of keloids and its potential value as a target for therapeutic intervention.

From the perspectives of single-cell transcriptomics and ligand–receptor interactions, chemokines produced by highly expressed fibroblast populations in keloids can continuously recruit infiltrating macrophages, T cells, and mast cells. In turn, TGF-β, IL-4/13, IL-17, and IL-6 secreted by M2 macrophages, Th2/Th17 cells, and mast cells further promote the proliferation and activation of fibroblasts and myofibroblasts as well as ECM synthesis (32). This bidirectional signaling between immune cells and fibroblasts establishes a self-sustaining chronic inflammatory microenvironment, providing a critical cellular basis for the persistent progression of keloids.

### 2.3 Other microenvironmental factors: vasculature, nerves, hypoxia, and metabolism

In addition to fibroblasts and immune cells, the local microenvironment of keloids also involves vascular-associated components and alterations in metabolic status, which may modulate lesion progression but are not the primary focus of this review. Existing studies indicate that endothelial dysfunction and hypoxic conditions can participate in local tissue remodeling, among which upregulation of HIF-1α is associated with angiogenesis, fibroblast proliferation, and enhanced collagen synthesis, and may synergize with pathways such as TGF-β/Smad and NF-κB to promote fibrotic responses (33). Meanwhile, metabolic stress alterations, including enhanced glycolysis and abnormal fatty acid metabolism, may support the highly synthetic state of keloid fibroblasts and participate in the regulation of related fibrotic programs (34).

Overall, hypoxia, vascular dysfunction, and metabolic reprogramming may play auxiliary regulatory roles in the keloid microenvironment, but their precise mechanisms remain to be further elucidated.

### 2.4 Integrated framework of key signaling pathways

The initiation and progression of keloids are not essentially driven by a single aberrant signaling event, but rather result from the interconnection and coordinated action of multiple profibrotic and inflammation-related pathways within the same tissue microenvironment. Among these, the TGF-β/Smad pathway serves as the central regulatory axis of fibrotic responses, not only upregulating the expression of ECM-related genes such as collagen, fibronectin, and CTGF, but also inhibiting ECM degradation through modulation of the MMP/TIMP balance, thereby promoting excessive ECM deposition and maintaining the activated myofibroblast phenotype. Meanwhile, a variety of inflammatory mediators can further amplify fibrotic effects through the JAK/STAT pathway; for example, IL-6–STAT3 promotes fibroblast proliferation and enhances resistance to apoptosis, whereas IL-4/IL-13–STAT6 facilitates Th2-skewed immune polarization and directly enhances collagen synthesis and myofibroblast differentiation. These inflammatory signals often act synergistically with the TGF-β pathway to jointly accelerate the progression of fibrosis. On the other hand, the tissue mechanical microenvironment can be coupled to the aforementioned fibrotic and inflammatory networks through multiple mechanisms. Cytoskeletal tension mediated by integrin–FAK and RhoA/ROCK, together with Ca²^+^ influx mediated by mechanosensitive ion channels such as PIEZO1, can convert mechanical stimuli including tissue tension, stretching, and matrix stiffness into intracellular biochemical signals. At the transcriptional level, these signals may further converge on mechanosensitive transcriptional axes such as Hippo–YAP/TAZ, enabling mechanical cues to directly regulate key biological processes including ECM synthesis, cell proliferation, and phenotypic remodeling. Existing studies suggest that Hippo–YAP/TAZ represents an important hub linking mechanical signaling to fibrotic responses (35). For the sake of an overall overview and to avoid repetition in subsequent sections, the key aberrantly activated molecular pathways involved in keloid initiation and progression are summarized in **Table 1**, and the following chapters will further discuss mechanical signaling, inflammatory responses, and their shared signaling hubs.

**Table 1:** Key molecular pathways abnormally activated in keloid initiation and progression.

Pathway	Role	Key references
TGF-β/Smad	Promotes ECM production and myofibroblast features, suppresses matrix degradation.	(36, 37)
Wnt (β-catenin/WNT5A)	Enhances proliferation and ECM synthesis, amplifies profibrotic signaling.	(38, 39)
Hippo–YAP/TAZ	Matrix stiffness and tension activate profibrotic transcriptional programs.	(40, 41)
Integrin–FAK and Rho–ROCK	Converts mechanical loading into contractile, matrix-depositing phenotypes.	(42–44)
JAK–STAT	Inflammation-driven transcriptional rewiring strengthens fibrotic responses.	(45–47)
NF-κB	Sustains chronic inflammation and a pro-fibrotic microenvironment.	(48–50)
NLRP3 inflammasome axis	Drives IL-1β/IL-18 output, sustaining inflammatory pro-fibrotic signaling.	(51–53)
Notch (Notch3)	Maintains fibroblast activation and remodeling programs.	(54–56)

## 3 Role of mechanical forces in keloid formation

**Figure 1:**
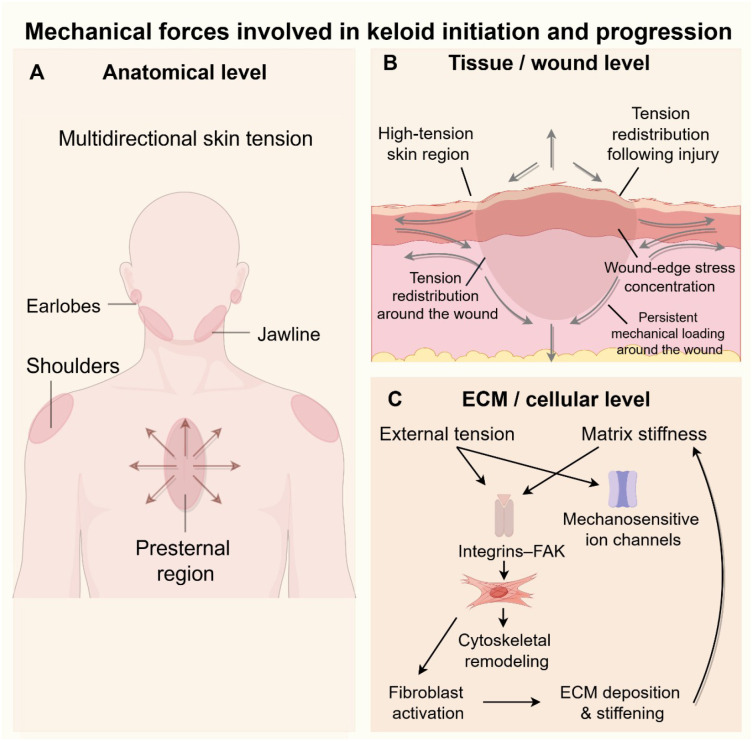
Mechanical forces involved in keloid initiation and progression. Mechanical factors contributing to keloid pathogenesis operate at three interconnected levels. **(A)** At the anatomical level, keloids preferentially develop in high-tension regions, such as the earlobes, jawline, shoulders, and presternal region. **(B)** At the tissue/wound level, injury induces local tension redistribution, wound-edge stress concentration, and persistent mechanical loading. **(C)** At the ECM/cellular level, external tension and matrix stiffness are transduced through integrins–FAK and mechanosensitive ion channels, promoting cytoskeletal remodeling, fibroblast activation, and excessive ECM deposition and stiffening. Together, these processes form a self-reinforcing mechanical loop that drives keloid initiation and progression. The figure was drawn by Figdraw and has been licensed for copyright.

Substantial basic and clinical evidence indicates that mechanical factors play a pivotal role in keloid development: lesions preferentially arise in high-tension regions, often extend outward in a “crab claw–like” pattern, and the high recurrence rate is closely related to incision design, suturing technique, and postoperative tension control. In recent years, studies employing mechanical measurements, finite element analysis, and cellular mechanobiology have further demonstrated that the mechanical microenvironment is a key driving force in the initiation and progression of keloids. These mechanical influences can be conceptualized across three interconnected levels—anatomical tension distribution, wound-edge stress redistribution after injury, and ECM/cellular mechanotransduction—which together shape keloid initiation and progression (**Figure 1**).

### 3.1 Distribution of skin tension and high-risk anatomical regions

Clinically, keloids preferentially develop in high-tension areas such as the presternal region, shoulders, lateral upper arms, auricle, and mandibular margin, but are uncommon in low-tension sites such as the palms and soles and the flexor surfaces of joints. Langer’s lines describe the predominant directions of skin tension, and keloids are more likely to occur in regions subjected to greater tensile forces. Accordingly, Ogawa et al. (2012) (57) proposed the “mechanical tension hypothesis,” positing that pathological scarring is a “tension-dependent disease.” In high-tension regions, persistent traction and shear during injury and healing are more likely to occur, facilitating keloid initiation, progression, and outward growth.

Finite element analyses support this view. Ishii et al. (2024) (58) performed finite element modeling of presternal keloids and found that lesions frequently extend outward in a “crab claw–like” pattern along the direction of tension, with stress concentration surrounding the lesion that coincides with the scar growth region. These findings indicate that high-tension regions per se constitute a risk factor for keloid development and recurrence, and that mechanical forces play a critical role in determining lesion morphology, orientation, and growth rate.

### 3.2 Tissue-level mechanical microenvironment and perilesional stress-concentrated regions

Keloids do not expand uniformly within the lesion; instead, they exhibit a centripetal-to-peripheral “invasive” pattern, forming a more elevated and erythematous zone of active hyperplasia at the margin. High-frequency ultrasonography and shear-wave elastography have shown marked increases in stress, strain, and stiffness in the perilesional area (59), creating a characteristic region of stress concentration. These regions closely coincide with the areas of peripheral extension, indicating a spatial concordance between local tensile forces and intracellular mechanosignaling.

Dohi et al. (2019) (60) found that perilesional stress and strain were significantly increased and closely associated with activation of caveolin-1/ROCK signaling, pathways that are strongly linked to scar progression. In addition, finite element analysis showed that stress-concentrated regions in acne-related keloids correspond to the clinically observed “crab claw–like” peripheral extension, suggesting that localized stress concentration may facilitate outward lesion growth.

### 3.3 Mechanosensing in fibroblasts and intracellular mechanotransduction

Fibroblasts primarily sense external mechanical cues through three key structural and functional modules: (1) the integrin–focal adhesion complex, (2) the cytoskeleton and intracellular tension system, and (3) mechanosensitive ion channels (61). When tissue tension or matrix stiffness increases, integrin-mediated cell adhesion is enhanced and activates FAK, which subsequently regulates actomyosin contraction, stress fiber assembly, and cytoskeletal remodeling through RhoA/ROCK signaling, thereby enabling cells to convert extracellular mechanical signals into a controllable intracellular mechanical state and, in turn, drive the reprogramming of downstream transcriptional programs (62–65).

At the level of transcriptional regulation, YAP/TAZ serve as core molecules linking mechanical stimulation to fibrotic effects: their nucleocytoplasmic shuttling is jointly regulated by matrix stiffness and intracellular tension, and they are more likely to translocate into the nucleus under conditions of high stiffness and high tension, thereby upregulating genes associated with myofibroblast differentiation and ECM synthesis (66, 67). Deng et al. (2021) (68) showed that YAP/TAZ expression was significantly elevated in keloid fibroblasts and was positively correlated with fibrotic markers such as α-SMA; inhibition of YAP/TAZ markedly reduced cellular proliferation and collagen synthesis, further supporting their critical role in mechanotransduction and the maintenance of fibrosis. Ross et al. (2024) (69) found, in a model with precise regulation of ECM elasticity and mechanical stress, that keloid fibroblasts exhibited abnormal lysosomal remodeling and enhanced exocytosis, suggesting that dysregulated control of ECM dynamic homeostasis may represent another important pathway by which mechanical signaling promotes fibrosis.

In addition to YAP/TAZ, mechanical stretch can also enhance the expression of profibrotic factors through pathways such as PI3K/Akt–GSK-3β/β-catenin and act synergistically with the TGF-β pathway, thereby further amplifying collagen synthesis and ECM remodeling (70).

### 3.4 Mechanical microenvironment and clinical evidence

The involvement of mechanical factors in keloid development is evident not only at the cellular and molecular levels but is also supported by clinical and translational studies. First, keloids commonly arise in high-tension regions, and finite element analyses have shown that the growth direction of acne-related keloids is parallel to the principal axis of thoracic tensile stress, with stress concentration at the lateral lesion margin corresponding to the clinically observed “crab claw–like” peripheral extension, further underscoring the relationship between traction magnitude and the pattern of lesion expansion.

Moreover, studies on intraoperative and postoperative tension management have shown that tension-reducing sutures and pressure therapy can markedly lower keloid recurrence rates. Early studies reported high recurrence after excision along high-tension lines alone, whereas the use of tension-reducing sutures effectively decreased recurrence; pressure therapy, commonly applied after burns and in some keloids, can significantly improve scar texture and stiffness by altering local stress distribution, with clear clinical benefits (71, 72).

In summary, mechanical forces exert multilayered effects during keloid pathogenesis, spanning lesion morphology and growth patterns in high-tension regions as well as mechanotransduction and gene-expression remodeling at the cellular level, collectively highlighting the critical contribution of mechanical cues to keloid progression. Clinical approaches such as tension-reducing sutures and pressure therapy further support the therapeutic potential of modulating mechanical forces as a form of effective intervention.

## 4 Role of inflammatory responses in keloid formation

**Figure 2:**
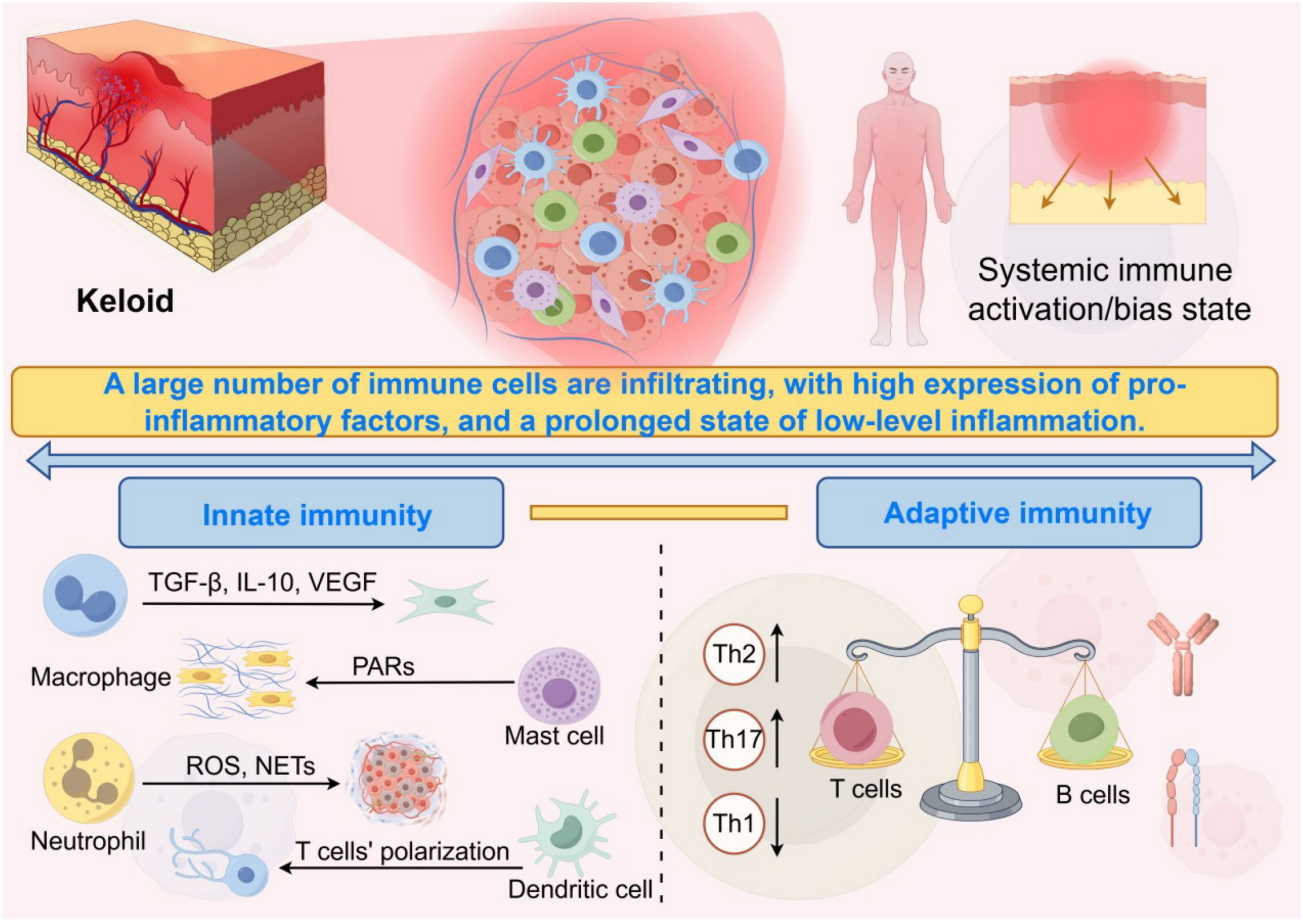
This image illustrates the role of the inflammatory response in the formation of keloids. The upper left part is magnified to show the characteristics of immune cell infiltration and collagen fiber proliferation in the keloid. The upper right corner indicates that keloid patients have systemic immune activation/bias. The lower part introduces the participation of innate and adaptive immunity in this inflammatory response. The figure was drawn by Figdraw and has been licensed for copyright.

A large body of evidence indicates that keloids are not only fibroproliferative lesions, but also a type of “inflammatory fibrotic lesion” characterized by chronic, mild, yet persistent inflammation. Unlike the self-limited inflammatory response observed during normal wound healing, patients with keloids often exhibit sustained immune cell infiltration, an imbalanced cytokine network, and prolonged activation of inflammatory signaling pathways, thereby maintaining the profibrotic phenotype of fibroblasts (**Figure 2**).

### 4.1 Transition from acute inflammation to chronic low-grade inflammation

During normal wound healing, inflammation rapidly resolves and transitions into the reparative phase; in keloids, however, wound-induced inflammation does not terminate within a short period but instead evolves into a prolonged low-grade inflammatory state characterized by persistent immune-cell accumulation and sustained overexpression of pro-inflammatory cytokines, keeping fibroblasts chronically exposed to an inflammation-driven microenvironment that delays repair and promotes lesion overgrowth and ECM accumulation (73).

Multiple reviews on the immune microenvironment have indicated that keloid tissues exhibit infiltration of macrophages, mast cells, dendritic cells, and various T-lymphocyte subsets, and that these cells collectively sustain persistent inflammation and drive fibrotic responses through the secretion of cytokines such as IL-4, IL-6, IL-10, IL-13, IL-17, TNF-α, and TGF-β (74). Transcriptomic analyses have further shown that the immune imbalance associated with keloids is not confined to the lesion itself, as similar alterations can also be observed in the clinically normal-appearing skin surrounding the lesion, suggesting the presence of systemic immune activation and immune skewing in affected individuals. Wu et al. (2020) (75) reported that Th2-related genes and JAK3-associated pathways were significantly upregulated in keloid tissues, accompanied by activation of Th1- and Th17-related pathways, resulting in broad dysregulation of immune regulation. Notably, similar immune-activation expression patterns could also be detected in unaffected skin, further supporting the possibility that patients with keloids may exhibit systemic immune deviation.

### 4.2 Innate immune cells: macrophages, mast cells, and others

As innate immune cells, macrophages and mast cells play important roles in the inflammatory microenvironment of keloids. Studies have shown a marked increase in M2 macrophages, which exhibit a profibrotic transcriptional phenotype resembling that of tumor-associated macrophages (76). M2 macrophages can stimulate fibroblast activation and ECM production by releasing TGF-β, IL-10, and VEGF (77). In addition to promoting angiogenesis, they also participate in immune regulation, thereby establishing a pro-fibrotic immune microenvironment.

In addition, the role of mast cells has also attracted increasing attention. Studies have shown that mast cell numbers are elevated and degranulation activity is enhanced, and the released histamine, proteases, and cytokines contribute to symptoms while also directly stimulating fibroblast proliferation and collagen production via protease-activated receptors (PARs) (78, 79). Moreover, mast cells are often located in close proximity to nerve fibers, and their secretory products participate in regulating fibrosis as well as symptoms such as pruritus and pain (80).

In addition, neutrophils and dendritic cells may also play roles in early keloid lesions; for example, the former can remodel the local microenvironment through reactive oxygen species (ROS) and neutrophil extracellular traps (NETs), whereas the latter may influence the balance of host immune responses by regulating T-cell polarization (81).

### 4.3 Adaptive immunity: dysregulation of T cells and B cells

Skewing of T-cell subsets is central to the inflammatory mechanisms of keloids. Transcriptomic and immunohistochemical studies show that keloid lesions display upregulated Th2- and Th17-associated markers with a relative paucity of Th1-related factors, indicating an adaptive immune bias dominated by Th2/Th17 responses. Th2 responses can enhance fibroblast proliferation and collagen synthesis via IL-4 and IL-13 while promoting macrophage polarization toward the M2 phenotype, whereas Th17 cells and their product IL-17A can exert profibrotic effects by recruiting neutrophils, amplifying inflammatory responses, and remodeling the ECM.

RNA-seq analyses have shown increased Th2-associated gene expression and enhanced JAK3 signaling in both keloid lesions and adjacent perilesional skin, consistent with clinical observations that the IL-4Rα antagonist dupilumab alleviates pruritus and may reduce scar activity in some keloid patients (82, 83). These observations further support the relevance of the Th2-associated IL-4/IL-13 axis and its downstream JAK/STAT signaling.

Regarding regulatory T cells (Tregs), studies indicate that an increase in Foxp3^+^ Tregs in keloids may reflect an immunosuppressive negative-feedback mechanism (84). However, Tregs may sometimes undergo functional reprogramming, whereby they not only suppress antifibrotic immune responses but also promote fibrosis. Research on B cells in keloids is limited, although some evidence suggests that B cells may modulate the immune microenvironment by producing antibodies or secreting cytokines.

### 4.4 Key inflammatory mediators and signaling pathways

The network of inflammatory cytokines and chemokines in keloids remains in a persistently activated state, among which Th2-related factors (IL-4, IL-13), the Th17-related factor (IL-17A), pro-inflammatory factors (IL-6, TNF-α), and the profibrotic factor TGF-β1 are the most representative driving molecules. Overall, these cytokines, on the one hand, sustain chronic low-grade inflammation through the polarization and recruitment of immune cells and, on the other hand, can act directly on fibroblasts and synergize with the TGF-β axis, thereby promoting ECM remodeling and fibrotic programs.

**Figure 3:**
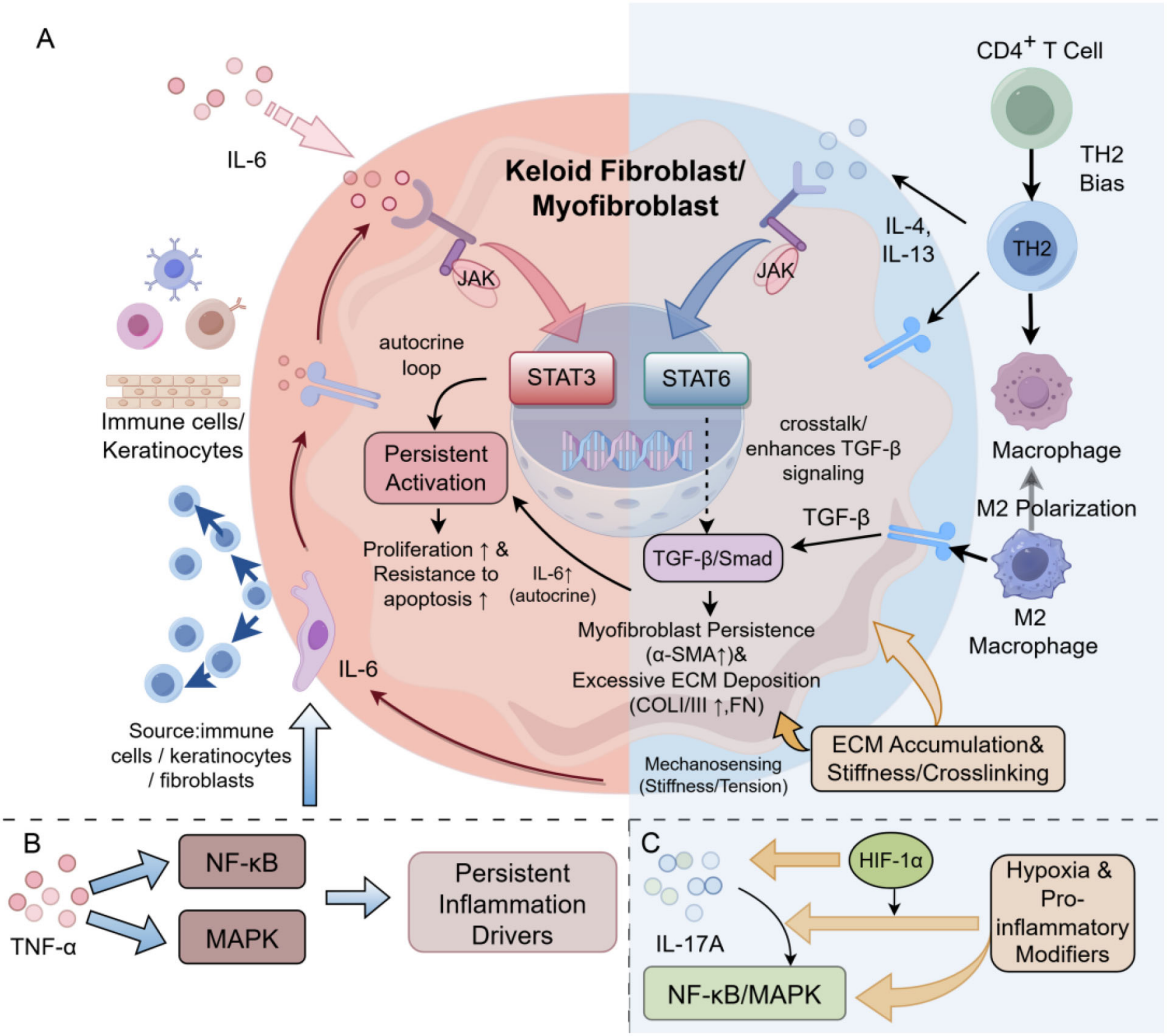
Key inflammatory and profibrotic signaling pathways in keloids. **(A)** IL-6 and IL-4/IL-13 signaling converge with TGF-β/Smad signaling in keloid fibroblasts/myofibroblasts, promoting persistent activation, myofibroblast persistence, and ECM accumulation/stiffness. Crosstalk with keratinocytes, Th2 cells, and M2 macrophages further reinforces this profibrotic state. **(B)** TNF-α activates NF-κB and MAPK signaling, contributing to persistent inflammatory drivers. **(C)** IL-17A, HIF-1α, and hypoxia-associated pro-inflammatory modifiers further enhance NF-κB/MAPK signaling, contributing to chronic inflammation and fibrosis. The figure was drawn by Figdraw and has been licensed for copyright.

In the Th2 axis, IL-4/IL-13–JAK/STAT6 not only reinforces Th2 immune skewing, but can also directly promote fibroblast proliferation, collagen synthesis, and differentiation into myofibroblasts (85, 86). In the maintenance of chronicity, IL-6–JAK/STAT3 is markedly upregulated in keloids, where it can enhance fibroblast proliferation and resistance to apoptosis, and contribute to the transition of inflammation toward a chronic state (87). In the Th17 axis, IL-17A can promote ECM remodeling and profibrotic transcriptional programs by amplifying inflammatory responses, enhancing IL-6- and HIF-1α-related signaling, and acting synergistically with TGF-β/Smad (88, 89). In addition, TNF-α can enhance the expression of inflammatory mediators and adhesion molecules through hubs such as NF-κB/MAPK, thereby contributing to the maintenance of chronic inflammation and the amplification of intercellular communication (**Figure 3**).

JAK/STAT and NF-κB are two major inflammatory signaling pathways in keloids, and cytokine-driven activation of JAK/STAT promotes T-cell polarization while regulating fibroblast proliferation, migration, and ECM synthesis. NF-κB is responsive to diverse upstream stimuli and remains constitutively activated. By inducing the expression of proinflammatory cytokines, chemokines, and adhesion molecules, it contributes to the maintenance of chronic inflammation (**Table 2**).

**Table 2:** Key inflammatory factors and signaling pathways.

Inflammatory factor/signaling pathway	Mechanism	References
IL-6-related inflammatory axis/JAK-STAT3	IL-6 activates STAT3 to promote fibroblast proliferation and collagen programs; STAT3 supports sustained cytokine and chemokine expression.	(90–92)
IL-1β/TNF-α-related inflammatory axis	IL-1β and TNF-α activate NF-κB and MAPK to induce inflammatory mediators and ECM remodeling, supporting immune infiltration and fibrosis.	(93–95)
IL-17-related inflammatory axis STAT3/HIF-1α	IL-17 enhances STAT3 and hypoxia-linked inflammatory programs, increasing fibroinflammatory activity and excessive ECM production.	(96–99)
IL-13-related inflammatory axis JAK-STAT6	IL-13 activates STAT6, enhances profibrotic signaling, and promotes collagen deposition; fibrosis in turn sustains Th2 skewing and IL-13 production.	(100, 101)
CCL2 chemokine axis CCR2 macrophages	CCL2 recruits CCR2-positive monocytes and macrophages; macrophage cytokines amplify fibroblast activation and collagen deposition.	(102–105)
CXCL12 chemokine axis CXCR4 TGF-β/Smad	TGF-β Smad signaling reshapes the chemokine milieu; CXCL12-CXCR4 recruitment sustains inflammatory infiltration and reinforces fibrosis.	(106–109)
Innate immunity pathway/TLR4-MyD88-NF-κB	Damage signals activate TLR4 MyD88 and NF-κB to elevate cytokines; the resulting inflammation strengthens profibrotic signaling.	(110, 111)
PI3K/Akt/mTOR	PI3K Akt mTOR enhances fibroblast growth and collagen synthesis; pathway activity also supports sustained inflammatory mediator production.	(112–114)

## 5 Mechanically dependent and mechanically independent sources of inflammation and their significance

The chronic inflammation associated with keloids is not driven by the repeated activation of a single mechanism, but is instead jointly sustained by both mechanically independent and mechanically dependent factors. Among these, mechanically independent drivers are mainly related to genetic susceptibility, systemic immune bias, and local hypoxia and metabolic stress within the lesion, all of which can maintain the immune response in a persistently overactivated state even when local tissue tension is not high. In contrast, mechanically related drivers are more commonly observed in regions with high tension or marked matrix stiffening: mechanical stimuli can be directly sensed by immune cells, triggering Ca²^+^ influx through mechanosensitive ion channels and subsequently activating inflammatory transcriptional pathways such as NF-κB; they can also induce mechanotransduction in fibroblasts, alter their secretory phenotype, and upregulate chemokines such as CCL2 and CXCL12, thereby promoting the recruitment and retention of immune cells within the lesion. In brief, the former establishes the baseline conditions for the persistent presence of inflammation, whereas the latter renders inflammation more likely to accumulate in mechanically abnormal regions and to interact with the fibrotic process in a mutually reinforcing, stepwise amplifying manner (115).

### 5.1 Inflammation drivers independent of mechanical cues: genetics, systemic immune bias, and hypoxic metabolism

The occurrence of certain inflammatory responses in keloids may not be entirely dependent on mechanical stimulation. Keloids exhibit familial aggregation and marked ethnic differences, suggesting that genetic susceptibility may place the host in a relatively immune-primed basal state at the level of immune regulation (116). Multiple transcriptomic studies have found that not only are immune-related pathways significantly upregulated within the lesion, but similar immune expression profiles can also be observed in adjacent skin that appears clinically normal, indicating that some patients may possess a systemic immune bias that is not directly induced by local mechanical factors (117). In addition, hypoxia and metabolic reprogramming within the lesion microenvironment may also constitute a relatively independent inflammation-driving pathway. By upregulating HIF-1α, hypoxia can drive cellular metabolism toward glycolysis and further promote the expression of multiple inflammation-related genes (118). This coupling of hypoxia and metabolism is essentially a form of microenvironmental stress response, and its degree of activation does not fully correspond to the magnitude of local tissue tension. Taken together, even in the absence of substantial mechanical stimulation, factors such as genetic susceptibility, systemic immune bias, and hypoxia-associated metabolic stress can still maintain the cytokine network at a relatively high level of activation, thereby providing a foundation for the subsequent amplification and persistence of mechanically related inflammation.

### 5.2 Mechanically induced inflammation: how mechanical signals are converted into immune activation and cellular recruitment

Unlike the aforementioned drivers of inflammation, the defining feature of mechanically induced inflammation is that mechanical signals themselves can directly trigger and sustain inflammatory responses. Within the high-tension, matrix-stiffened microenvironment characteristic of keloids, the conversion of mechanical signals into inflammatory output is mediated primarily through two pathways. The first pathway involves the direct sensing of mechanical stimuli by immune cells: increased matrix stiffness or elevated local tension can activate Piezo1 channels on the membranes of immune cells such as macrophages, inducing Ca²^+^ influx and initiating key inflammatory transcriptional pathways including NF-κB, thereby promoting the release of inflammatory mediators and sustaining a profibrotic immune phenotype (119). The second pathway involves stromal cell–mediated recruitment of immune cells through mechanical responsiveness: when fibroblasts are exposed to stretch or high-tension stimulation, their YAP/TAZ-related mechanotransduction pathways are activated, their secretory profile is remodeled, and the expression of chemokines such as CCL2 and CXCL12 is upregulated, thereby enhancing the migration and retention of immune cells in the lesion area (120–123). The synergistic action of these two pathways renders high-tension regions more prone to developing persistent inflammation and establishes a local positive feedback loop that mutually reinforces the fibrotic process.

Overall, mechanically independent inflammation provides the lesion with an immune baseline state that is readily activated and prone to persistence. By contrast, mechanically induced inflammation offers a compelling explanation for why inflammation preferentially accumulates in regions of high tension and matrix stiffening, where it exhibits greater persistence and a stronger profibrotic amplifying effect. These two inflammatory driving mechanisms often coexist and overlap within the same lesion, and their synergistic interaction may constitute a key mechanistic basis for the progressive nature and high recurrence tendency of keloids.

## 6 Interactions between mechanical forces and inflammatory responses and the positive feedback loop

**Figure 4:**
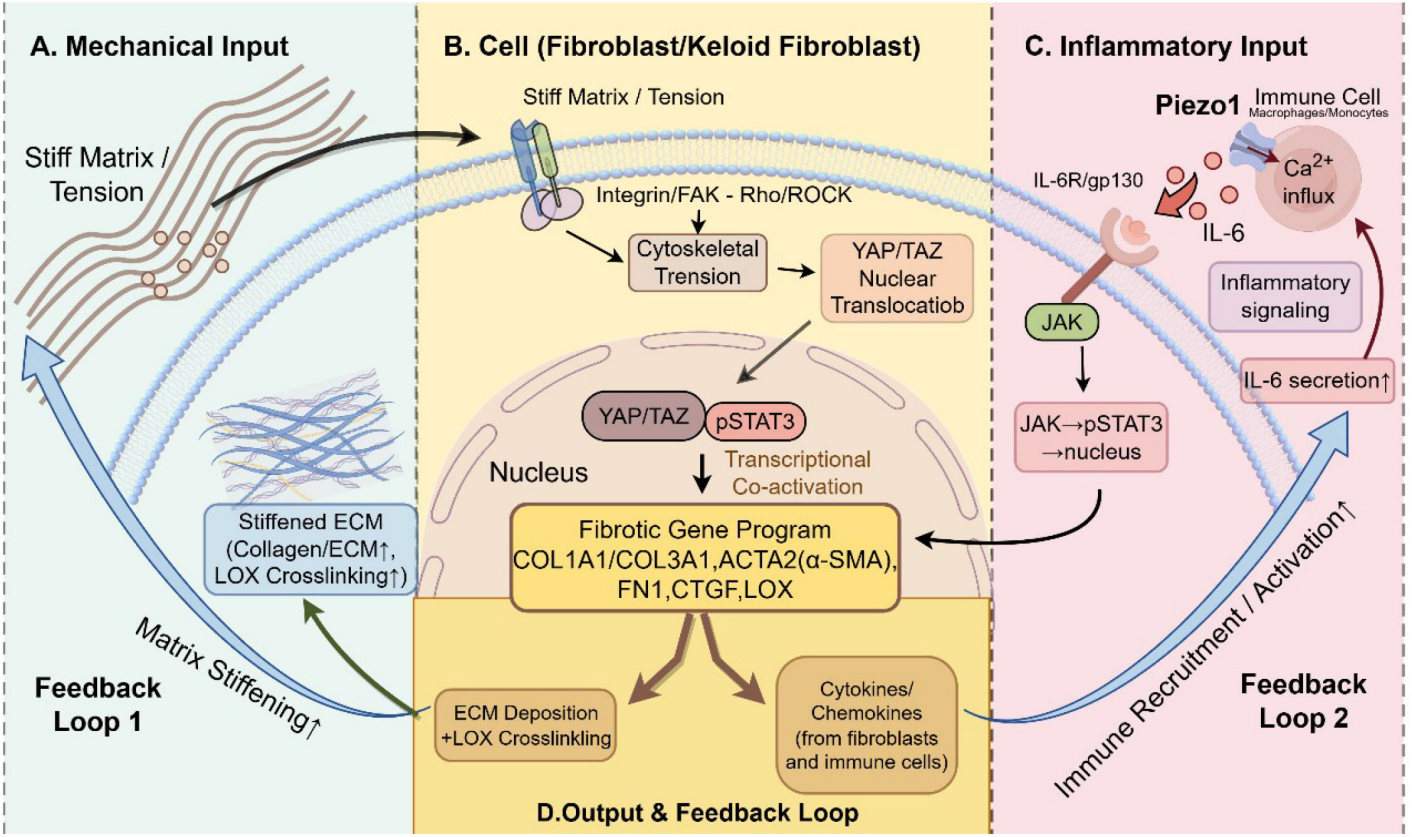
Mechanotransduction and inflammatory signaling are coupled to form a self-amplifying feedback loop that drives the fibrotic gene program in fibroblasts/keloid fibroblasts. **(A)** Mechanical input: matrix stretching and stiffening are sensed by cells, while excessive ECM deposition and LOX-mediated crosslinking further increase matrix stiffness, forming feedback loop 1. **(B)** Cellular response: mechanical stimulation activates integrin/FAK–Rho/ROCK signaling, increases cytoskeletal tension, and promotes YAP/TAZ nuclear translocation. In the nucleus, YAP/TAZ cooperates with pSTAT3 to induce profibrotic gene expression, thereby enhancing ECM deposition, crosslinking, and fibroblast- and immune-derived mediator production. **(C)** Inflammatory input: inflammatory cues activate Piezo1-mediated Ca²^+^ influx in immune cells and stimulate IL-6R/gp130–JAK–STAT3 signaling, promoting inflammatory and profibrotic transcriptional responses and further IL-6 production. **(D)** Output and feedback: fibroblast-derived mediators recruit and activate immune cells, forming feedback loop 2, which sustains chronic inflammation and amplifies fibrotic remodeling. The figure was drawn by Figdraw and has been licensed for copyright.

The aberrant healing observed in keloids is not the result of mechanical factors or inflammatory factors acting alone. More commonly, local high tension and matrix stiffening provide the lesion with a sustained physical driving force, whereas chronic inflammation makes this process more likely to be maintained and amplified. In recent years, cross-disciplinary research integrating mechanobiology and immunology has increasingly suggested that mechanical signals can not only alter local vascular and cellular behavior, thereby influencing the manner and extent to which immune cells infiltrate the lesion, but that inflammation can in turn directly reshape the mechanical properties of the tissue by affecting ECM production, degradation, and crosslinking, ultimately forming a self-reinforcing loop in which mechanical forces, inflammation, and fibrosis mutually drive one another forward (**Figure 4**). Evidence from animal models also supports the view that this mutually amplifying relationship plays a critical role in the progression of fibrosis (124, 125).

### 6.1 Mechanical stress–driven inflammatory activation

At the vascular and endothelial levels, excessive stretch or increased matrix stiffness can directly induce an inflammation-like activation state in endothelial cells. Nonphysiological shear stress and tensile forces enhance the inflammatory phenotype of endothelial cells, upregulating adhesion molecules such as ICAM-1 and VCAM-1 as well as proinflammatory mediators including IL-6 and TNF-α, thereby promoting leukocyte adhesion and transendothelial migration, which makes local inflammation more likely to occur and persist (126–129). The importance of this process lies in the fact that it provides a relatively “upstream” pathway through which the mechanical microenvironment can continuously drive immune-cell infiltration into the lesion; that is, mechanical abnormalities not only act on cells within the lesion itself, but can also persistently promote the recruitment of immune cells into the lesional microenvironment through the vascular wall.

### 6.2 Inflammation-mediated ECM remodeling and alterations in matrix mechanics

In addition to serving as a downstream target of mechanical stimulation, inflammatory processes can also influence the synthesis, degradation, and homeostatic balance of the ECM, thereby altering the mechanical properties of the local matrix. A variety of pro-inflammatory and pro-fibrotic cytokines can enhance ECM component production in fibroblasts while simultaneously inhibiting matrix degradation, for example by reducing matrix metalloproteinases (MMPs) and increasing tissue inhibitors of metalloproteinases (TIMPs), thus promoting ECM accumulation and progressive stiffening (130). Meanwhile, inflammation can also promote the expression of lysyl oxidase (LOX) and related collagen cross-linking enzymes, thereby increasing collagen cross-linking and further enhancing matrix stiffness (131).

### 6.3 Closed-loop hubs and shared nodes

Within this self-amplifying loop, several shared signaling nodes link mechanical input to inflammatory and profibrotic output. Increased local tension and matrix stiffening activate mechanosensitive pathways such as integrin–FAK and promote the nuclear activity of YAP/TAZ, whereas inflammatory mediators such as IL-6 transmit inflammatory signals through the JAK/STAT pathway. These signals converge at the transcriptional level to enhance fibrosis-related gene expression, thereby promoting persistent ECM deposition, excessive cross-linking, and progressive matrix stiffening. In turn, the stiffened and chronically inflamed microenvironment further reinforces mechanotransduction and immune activation. Among the shared nodes involved in this process, Piezo1 and YAP/TAZ are currently the best-characterized examples in keloids, whereas TRPV4 and integrin–FAK may also participate in this coupling process. Among the key nodes described above, Piezo1 is positioned closer to the upstream entry point of the loop, where it can rapidly convert mechanical changes such as matrix stiffening and mechanical stretch into intracellular signals and initiate inflammation-related programs (132, 133). Mechanical stimulation can activate the opening of Piezo1 channels and mediate Ca²^+^ influx, thereby activating inflammatory transcriptional axes such as NF-κB (or coupling with inflammasome-related pathways) and promoting the synthesis and release of inflammatory mediators such as IL-6 (134, 135). This process converts mechanical signals into diffusible inflammatory signals with amplification potential, thereby further regulating the activation and interactions of surrounding immune cells and stromal cells. In doing so, it establishes a persistent and stable cytokine microenvironment that provides continuous upstream driving forces for the nuclear transduction of inflammatory signals and their convergence with mechanical signaling.

Unlike Piezo1, which primarily functions in upstream mechanotransduction, YAP/TAZ are more likely to serve as intranuclear hubs of the loop, directly determining whether mechanical signals can be stably converted into transcriptional output (136). Matrix stiffening and increased cytoskeletal tension can induce YAP/TAZ dephosphorylation and nuclear translocation, thereby enabling them to cooperate with transcription factors such as TEAD to regulate ECM synthesis and the maintenance of pro-fibrotic cellular phenotypes. At the same time, inflammatory mediators such as IL-6 promote STAT3 phosphorylation and nuclear translocation via JAK, allowing inflammatory and mechanical signals to converge spatially within the nucleus. Existing studies suggest that YAP/TAZ and STAT3 can act synergistically at the transcriptional level, jointly reinforcing the activation and sustained expression of fibrosis-related gene programs. When this intranuclear synergistic effect persists over time, ECM deposition and cross-linking are further increased, leading to sustained elevation of tissue stiffness, which in turn enhances YAP/TAZ nuclear localization and upstream mechanosensory processes. In this way, the feedback loop is reconnected to its initiating step, allowing the entire pathway to form a closed circuit and undergo continuous self-amplification.

### 6.4 The “mechanical force–inflammation–fibrosis” positive-feedback model

Under conditions of persistent inflammation, the balance between ECM synthesis and degradation is further disrupted, leading to massive collagen deposition, increased crosslinking, and a sustained rise in tissue stiffness. These changes, in turn, allow mechanical signaling to be continuously amplified and self-maintained. Matrix stiffening not only directly reinforces mechanotransduction, but also increases the sensitivity of local cells to inflammatory signals, thereby enabling inflammation and fibrosis to mutually promote and become locked within the same microenvironment. Ultimately, the lesion is unable to spontaneously enter a stable regression phase and instead exhibits marked persistent progression and a high tendency toward recurrence. This mechanistic model suggests that the core pathological feature of keloids does not lie in the abnormality of any single factor, but rather in the formation of a closed loop among multiple interacting factors that continuously reinforce one another. Therefore, reducing wound-edge tension and alleviating local stress concentration may simultaneously weaken both inflammatory initiation and mechanical driving effects. At the same time, interventions targeting the shared hubs between mechanical signaling and inflammatory responses, as well as key inflammatory signaling axes, may be more likely to mechanistically disrupt their coupling and thereby provide a theoretical basis for combined clinical treatment strategies.

## 7 Challenges in the treatment of keloids and research advances from the perspectives of mechanical forces and inflammation

### 7.1 Current therapeutic landscape and major challenges

**Figure 5:**
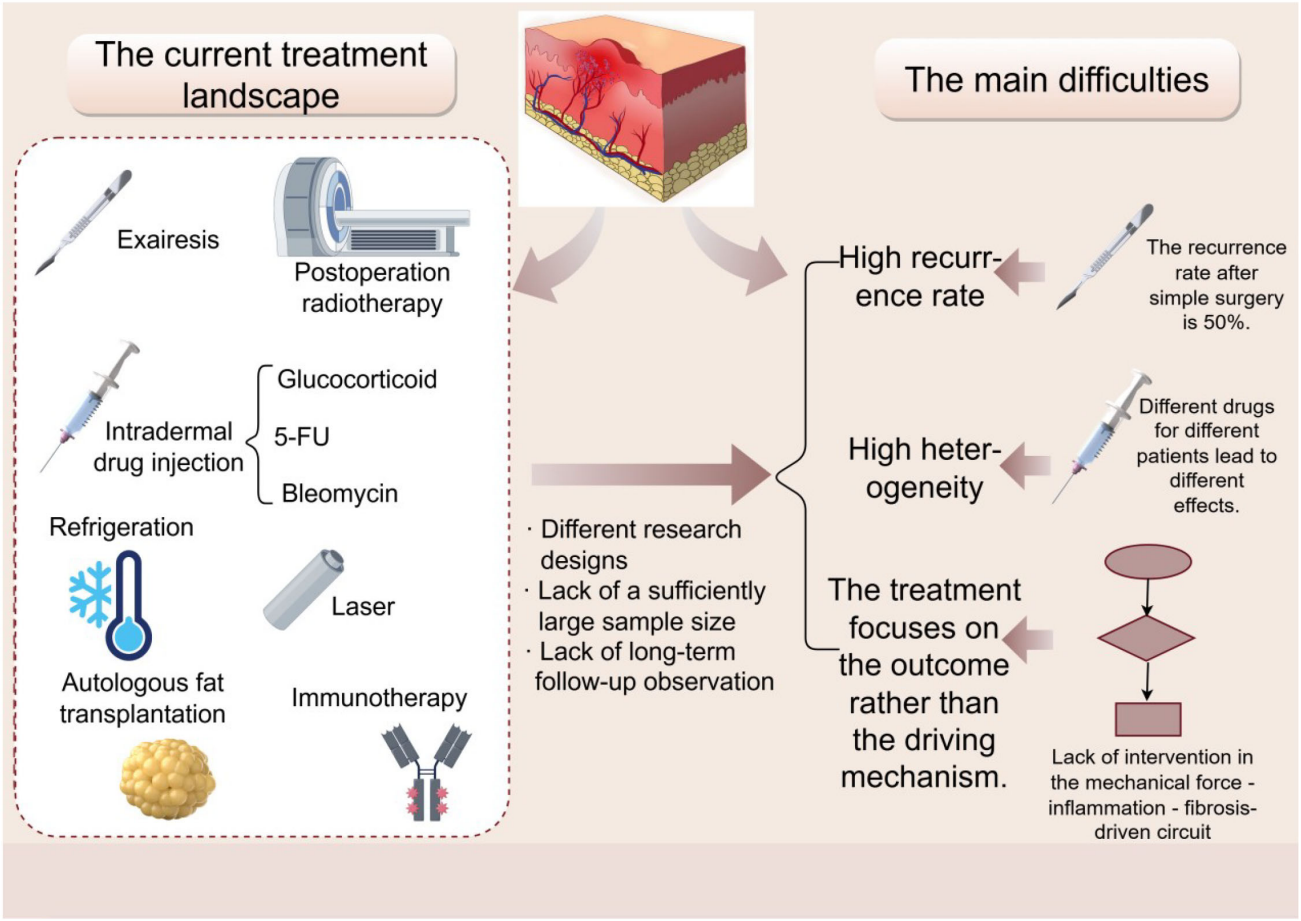
Current treatment methods for keloids include surgical excision, postoperative radiotherapy, intradermal drug injection (such as glucocorticoids, 5-FU, bleomycin, etc.), cryotherapy, laser therapy, autologous fat transplantation, and immunotherapy. Due to differences in research design and the lack of large enough samples and long-term follow-up observations, there are several major difficulties in clinical practice: (1) High recurrence rate: The recurrence rate of simple surgical treatment is over 50%. (2) High heterogeneity: The effects of different injected drugs or combined regimens vary among different patients. (3) Treatment methods focus on “results” rather than “driving mechanisms”: Current treatments still lack intervention in the “mechanical force - inflammation - fibrosis” driving loop. The figure was drawn by Figdraw and has been licensed for copyright.

Current treatments for keloids include surgical excision, postoperative radiotherapy, intralesional injections (e.g., corticosteroids, 5-fluorouracil [5-FU], bleomycin), cryotherapy, laser therapy, and pressure therapy, as well as a range of emerging pharmacologic and physical interventions (e.g., local botulinum toxin type A, autologous fat grafting, and immuno/gene therapies) (137). A recent systematic review suggests that the most commonly recommended first-line approach is silicone gel or silicone sheeting combined with intralesional corticosteroid injection, with optional adjunctive intralesional 5-FU, bleomycin, or verapamil. Other modalities, such as radiotherapy and laser therapy, can also be incorporated into combination regimens. Combination strategies are generally superior to any single modality (138); however, heterogeneity in study designs, along with insufficient sample sizes and limited long-term follow-up, precludes the establishment of a unified treatment algorithm. In clinical practice, several major difficulties and challenges remain:

1. High recurrence rate: the recurrence rate after surgery alone exceeds 50% (139), and even when combined with radiotherapy, intralesional injections, and pressure-reducing therapy, recurrence remains common in certain high-tension anatomical sites (140), indicating that current approaches do not fundamentally suppress the underlying pathogenesis.
2. High heterogeneity: keloid growth patterns and responses to available therapies vary substantially across lesion sites and among individuals, making it difficult to define a standardized treatment regimen. Reviews indicate that clinical outcomes differ widely between patients receiving different intralesional agents or combination strategies.
3. Current treatments emphasize “outcomes” rather than “driving mechanisms”: most available interventions physically remove or mitigate established scars (e.g., excision, cauterization, and compression), while targeted disruption of the mechanics–inflammation–fibrosis loop remains limited, resulting in variable efficacy and a lack of curative benefit. Although postoperative radiotherapy or injections have been reported to reduce short-term recurrence in facial lesions, robust evidence demonstrating sustained long-term efficacy through modification of the underlying pathological mechanisms is still lacking (**Figure 5**).

In summary, high-quality, mechanism-based, and durably effective therapies for keloids are still lacking. Therefore, improving the level of clinical evidence, refining combination treatment strategies, and developing new interventions grounded in pathophysiological mechanisms should be key priorities for future research and clinical practice.

### 7.2 Overview of conventional and multimodal treatment strategies

Multimodal regimens commonly used in practice include combinations such as “surgery + radiotherapy/intralesional injection + physical therapy”: for moderate-to-severe or recurrent lesions, wide excision with tension-reducing closure is performed, followed by adjuvant radiotherapy and intralesional corticosteroid injections; for small-volume lesions, local excision is combined with pressure earrings or silicone sheeting. Systematic reviews and meta-analyses indicate that triamcinolone acetonide (TAC) combined with 5-FU or bleomycin is more effective than TAC alone and provides better control of recurrence (141).

Physical modalities such as laser therapy, cryotherapy, silicone gel, and pressure garments are also recommended as important adjunctive treatments, as they can improve scar thickness, texture, and pigmentation and thereby enhance overall therapeutic efficacy. For example, at 12 months of follow-up, laser therapy combined with intralesional TAC injection showed advantages over TAC alone in improving scar outcomes and reducing recurrence.

### 7.3 Intervention strategies targeting mechanical forces

An increasing number of studies have shown that the formation and recurrence of keloids are closely associated with areas of stress concentration and persistent tensile forces. Therefore, the core objective of mechanical interventions is to reduce tension at the wound edge and minimize the sustained mechanical stimulation caused by a stiffened matrix as much as possible. Tension-reducing suturing techniques, including deep fascial or subcutaneous tension reduction and stepwise layered tension reduction, can transfer and disperse superficial tensile stress to deeper tissues, thereby alleviating the persistent mechanical load at the wound edge. Through appropriate design of incision direction or by adjusting the distribution of tension lines using flap-rearrangement techniques such as Z-plasty, excessive local traction can be reduced, thereby lowering the risk of recurrence (142). These practices are largely supported by clinical experience, retrospective studies, and some controlled studies, suggesting that they are particularly important in high-tension regions. Their limitations lie in the substantial variability in operative details, strong site dependence, and the difficulty of fully controlling confounding factors in research settings.

The second category of approaches involves exogenous tension reduction and mechanical unloading. Tape fixation, silicone sheets or silicone gel, and pressure therapy (such as pressure earrings/clips and pressure garments) are commonly used to prolong the tension-reducing effect, minimize repetitive minor traction stimuli, and improve scar appearance and symptoms to a certain extent. Overall, these methods have shown benefits, but their efficacy varies considerably. Their main limitations are a high dependence on patient adherence, marked interindividual variability, and generally limited effectiveness in improving mature scars.

The third category of approaches involves modulation of the overall mechanical stress environment of the wound. Negative-pressure wound therapy (NPWT) can reduce lateral tension across the incision, optimize local stress distribution, and promote incision healing during postoperative wound management, thereby demonstrating potential for application in scar prevention and treatment (143). Based on the current evidence, most relevant studies have focused on wound management and scar prevention, whereas direct evidence specifically addressing the control of keloid recurrence remains limited. Its limitations lie in the lack of unified standards regarding indications, duration of use, and treatment parameters.

**Figure 6:**
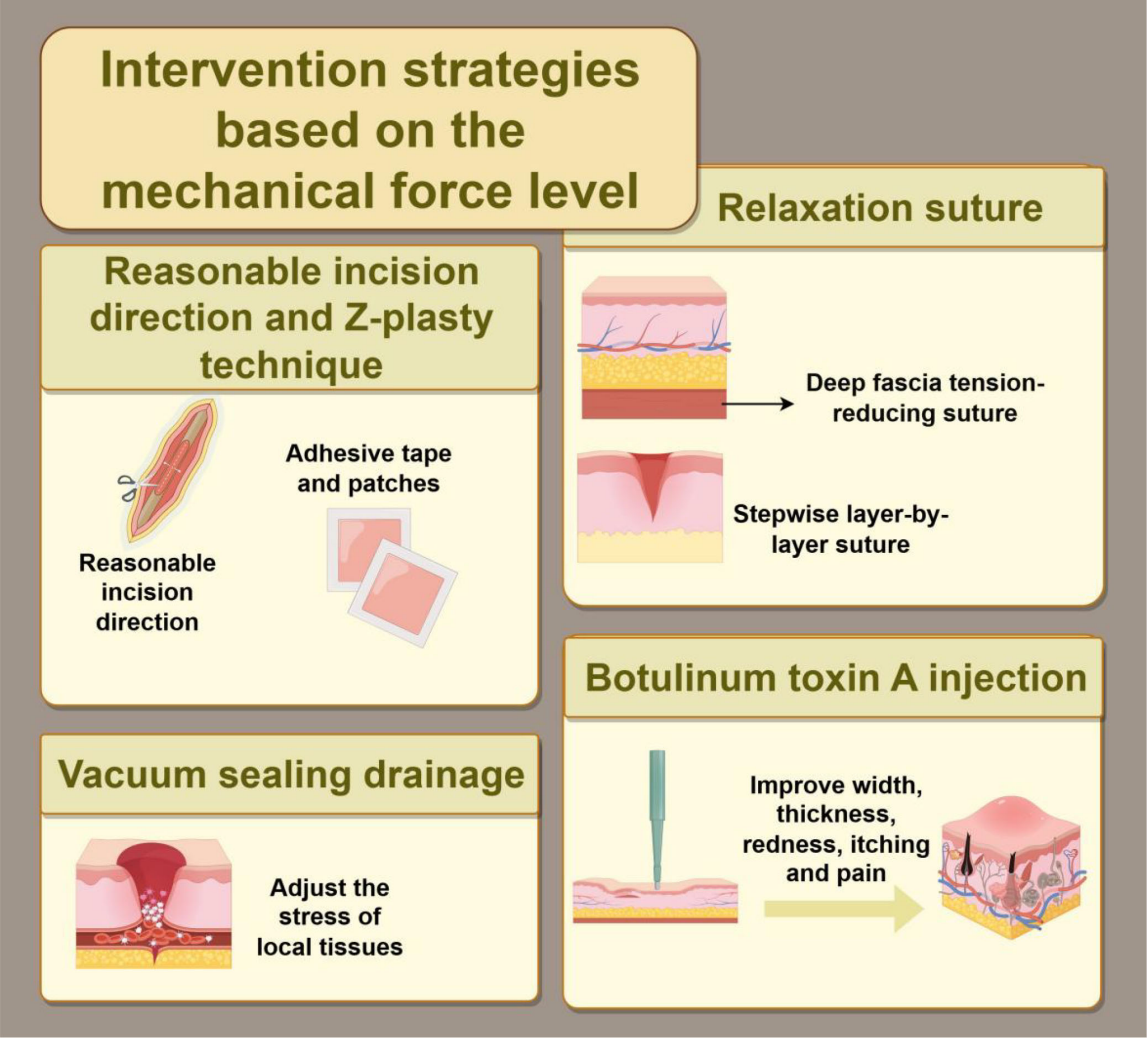
Intervention strategies based on mechanical force include: (1) tension-reducing suturing (deep fascia tension-reducing suturing, layer-by-layer stepwise tension reduction). (2) Reasonable incision direction, and Z-plasty (as well as adhesive tape, silicone patches). (3) NPWT (regulating local tissue stress conditions). (4) Botulinum toxin A injection (improving scar width, thickness, redness, itching and pain). The figure was drawn by Figdraw and has been licensed for copyright.

The fourth category of approaches aims to reduce dynamic traction. Local injection of botulinum toxin type A can attenuate the repetitive dynamic traction caused by muscle contraction, thereby providing a relatively low-tension healing environment for the wound edge, while also potentially exerting regulatory effects on inflammatory and fibrotic signaling pathways. Some studies have suggested that it can improve scar width, thickness, erythema, swelling, pruritus, and pain-related symptoms (144, 145). This approach has been supported mainly by small-sample clinical studies and case series, with encouraging preliminary efficacy, but further validation through large-sample studies with long-term follow-up is still required. Its limitations include substantial variability in injection dose, site selection, and treatment window, and it is not applicable to all anatomical locations (**Figure 6**).

### 7.4 Advances in targeted therapies based on inflammatory and immune pathways

The goal of anti-inflammatory and immunomodulatory therapies is to reduce the sustained input of cytokine signaling, inhibit the aberrant activation of fibroblasts, and diminish the promoting effects of the inflammatory microenvironment on ECM production and crosslinking.

Intralesional corticosteroid injection remains the current first-line foundational therapy and can alleviate scar hyperplasia by suppressing inflammatory factor expression, inducing fibroblast apoptosis, and reducing collagen synthesis. This therapy is widely used in clinical practice and is supported by relatively substantial evidence; however, its limitations include a still relatively high recurrence rate and the frequent occurrence of local adverse effects such as skin atrophy, telangiectasia, and pigmentary abnormalities, requiring a careful balance between efficacy and safety in clinical application.

Antimetabolic and antiproliferative agents are often used to improve the overall control of scars. 5-FU can interfere with DNA and RNA synthesis in fibroblasts, thereby inhibiting cell proliferation and promoting apoptosis (146). When combined with corticosteroids or bleomycin, 5-FU can further enhance scar volume reduction and recurrence control (147). Multiple systematic reviews and meta-analyses support the superiority of combination therapy over monotherapy. However, its limitations are also evident, as local adverse effects such as ulceration, pain, pigmentary abnormalities, and skin atrophy are not uncommon. In addition, substantial differences among studies in dosing frequency, dosage, and treatment duration result in poor reproducibility of treatment protocols.

**Figure 7:**
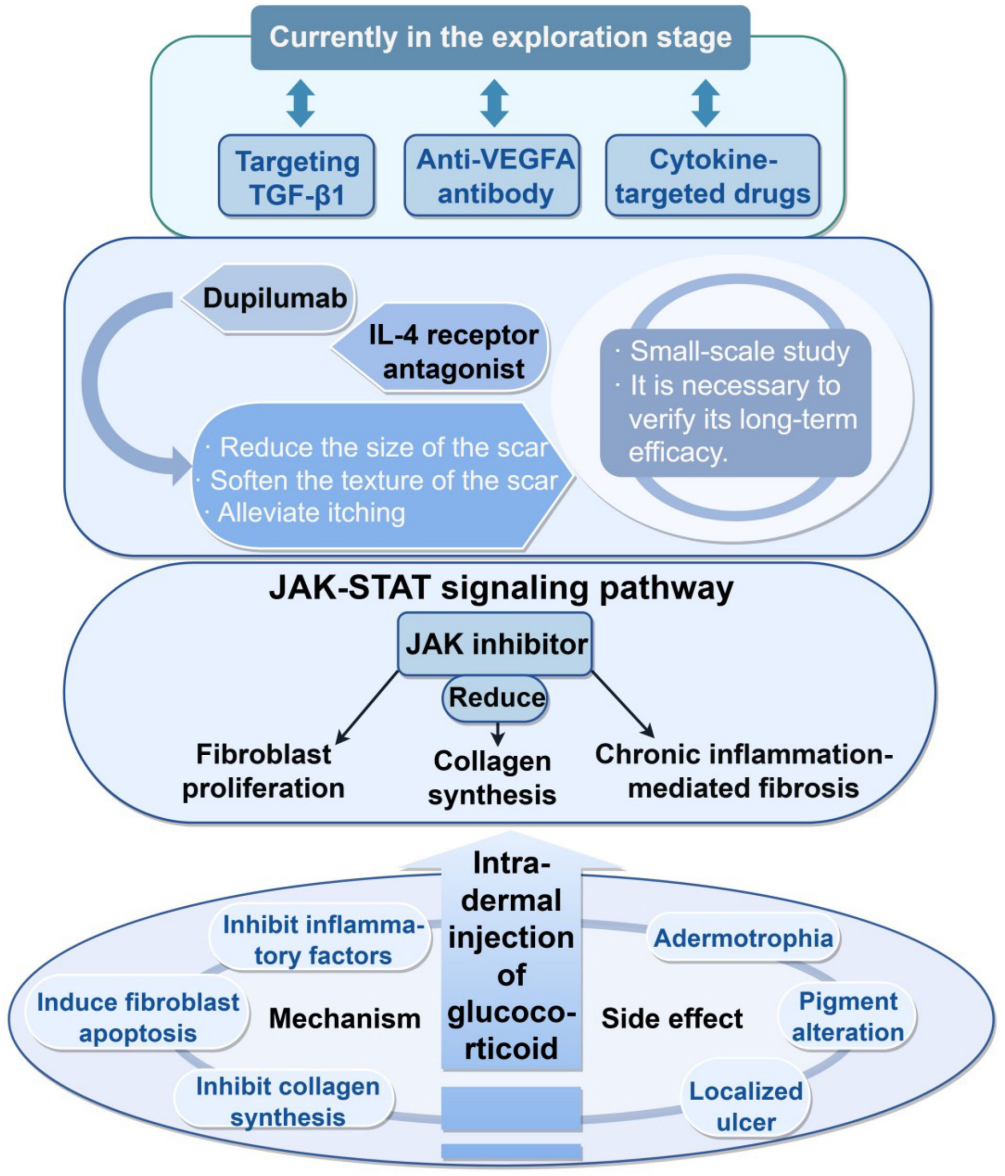
The progress of targeted therapy includes: (1) intralesional injection of glucocorticoids. The mechanism of action is to inhibit inflammatory factors, induce fibroblast apoptosis, and inhibit collagen synthesis, thereby reducing scar hyperplasia. Side effects include skin atrophy, pigmentation changes, and local ulcers. (2) JAK inhibitors can down-regulate the proliferation of fibroblasts and collagen synthesis, and may prevent fibrosis mediated by chronic inflammation. (3) Dupilumab, as an IL-4 receptor α antagonist, can reduce scar volume, soften the texture of scars, and alleviate symptoms such as itching. However, these studies are mainly small-scale research and case reports, and their long-term efficacy needs to be further confirmed. (4) Targeting TGF-β1, anti-VEGFA antibodies, etc. are in the exploration stage. The figure was drawn by Figdraw and has been licensed for copyright.

Emerging targeted therapies directed at immune pathways are still in the exploratory stage. As a key regulatory node of inflammation and immune cell function, the JAK/STAT pathway has been shown in existing studies to mediate the inhibitory effects of JAK inhibitors on the proliferation and collagen synthesis of keloid fibroblasts, and agents such as tofacitinib have demonstrated a certain degree of efficacy in individual cases. Targeted therapies against the IL-4/IL-13 axis, such as dupilumab, have been reported in a limited number of cases to improve scar volume, texture, and pruritus, and their benefits may be more pronounced in patients with Th2 skewing or concomitant atopic dermatitis (148). At present, these therapies are supported mainly by *in vitro* experiments, small-sample clinical studies, or case reports, and high-quality randomized controlled trials and long-term follow-up data are still lacking. Their target populations, optimal timing of intervention, and long-term safety all remain to be further clarified. In addition, therapeutic strategies targeting molecules such as TGF-β1 and VEGFA are also still under investigation, and they may provide new directions for future treatment (149) (**Figure 7**).

### 7.5 Synergistic interventions using an integrated “mechanical–inflammatory” combination approach

From a mechanistic perspective, merely suppressing the inflammatory response or solely reducing local tension is often insufficient to prevent the signal feedback loop from being reactivated at other points. Therefore, a more precise therapeutic strategy is to target the shared key nodes that simultaneously mediate mechanical signal input and amplify inflammatory and fibrotic effects, thereby reducing at the source the coupling between mechanical signaling and inflammatory/fibrotic pathways.

The shared nodes currently attracting attention can be broadly divided into three categories. The first category comprises integrin-associated adhesion signaling axes, such as FAK and its downstream pathways including ROCK, which are involved not only in cellular sensing of matrix stiffness and tensile forces but also in the regulation of inflammation-related transcription and cell migratory behavior. The second category includes nuclear mechanotransduction hubs, such as YAP/TAZ and their associated regulatory networks, which can convert matrix stiffening and cytoskeletal tension into sustained profibrotic transcriptional programs and act synergistically with inflammatory signaling to amplify these effects. The third category consists of mechanosensitive ion channels, such as Piezo1 and TRPV4, which can translate mechanical stimuli into Ca²^+^ signaling and downstream inflammatory transcriptional responses, thereby converting mechanical input more directly into inflammatory output. At present, research on these targets in keloids is still mainly based on *in vitro* studies, organoid models, or animal experiments, and clinical evidence remains very limited. Current limitations include unresolved issues related to drug delivery methods, long-term local safety, potential effects on normal wound repair processes, and patient stratification strategies; therefore, in the short term, these targets are more suitable as priorities for mechanistic validation and translational development rather than as direct replacements for existing clinical therapies.

Based on the above rationale, a more appropriate combination treatment strategy is to organically integrate mechanical environment modulation with anti-inflammatory and anti-proliferative therapies. In the early stage of injury, the priority should be to strengthen tension reduction and stress management in order to minimize the triggering of mechanical signaling and the initiation of inflammation as much as possible. During the high-risk phase of scar hyperplasia, local injection and related approaches may be used to suppress inflammatory responses and abnormal proliferation, and if there is clear evidence of immune bias, the corresponding targeted therapies may be combined. In the future, if targeted drugs against shared nodal hubs gain higher-level evidence supporting their safety and efficacy, they may be incorporated into the overall treatment regimen as important means of interrupting the pathogenic feedback loop. At the same time, study design should incorporate multi-omics stratification and risk-stratification strategies to clarify individual heterogeneity and improve the predictability of therapeutic outcomes.

## 8 Discussion and outlook

As a chronic inflammatory fibroproliferative disorder, keloids arise from the combined effects of the mechanical microenvironment, persistent inflammatory responses, and interconnected fibrotic signaling networks. This review summarizes recent advances in the pathogenesis, clinical characteristics, and therapeutic research of keloids, with particular emphasis on the roles of mechanical stress and inflammatory responses in their initiation and progression. We further propose an integrated therapeutic concept targeting the “mechanics–inflammation–fibrosis positive feedback loop.”

From a pathophysiological perspective, keloids are characterized not only by sustained activation of fibroblasts and myofibroblasts with excessive extracellular matrix deposition, but also by marked cellular heterogeneity and dysregulated signaling pathways. The literature has identified multiple fibroblast subpopulations (e.g., proliferative, inflammatory, and mechanosensitive phenotypes) and an immune microenvironment dominated by M2 macrophages, mast cells, and Th2/Th17 cells, providing a basis for viewing keloids as a form of “tumor-like fibrosis.” Studies have also quantified mechanical forces and demonstrated that “mechanical hotspots” in high-tension regions and along lesion margins exhibit increased tissue stiffness and stress, and that fibroblasts sense and amplify these mechanical cues through multiple mechanisms to drive fibrotic initiation and progression. The inflammatory profile of keloids reflects an uncontrolled transition from acute inflammation to persistent, low-grade chronic inflammation, thereby exacerbating fibrosis.

In summary, this article proposes a “mechanics–inflammation–fibrosis positive feedback loop” model. Under conditions of high tension and matrix stiffening, mechanoreceptor activation triggers cellular responses and inflammation; in turn, inflammatory cells and mediators promote progressive ECM deposition, while the subsequently stiffened matrix further enhances mechanotransduction and immune-cell polarization, together establishing a self-reinforcing loop. This framework not only helps clarify the principles governing keloid initiation and progression, but also provides a conceptual foundation for future therapeutic strategies.

However, substantial challenges remain in current research. The pronounced heterogeneity of keloids has not been adequately addressed, and most studies still treat keloids as a single entity. Long-term follow-up studies tracking the natural course of keloid development are lacking. Moreover, animal models and *in vitro* systems differ substantially from the *in vivo* context and therefore cannot fully recapitulate the true biological state of keloids. Finally, the causal relationships within the mechanics–inflammation interplay remain unclear, and identifying effective ways to disrupt this positive feedback loop represents a key direction for future efforts.

In addition, existing studies suggest that the skin or gut microbiota may participate in the initiation and progression of keloids by influencing immune homeostasis, chronic inflammation, and ECM remodeling; however, the current evidence is largely indirect and correlational, and a causal relationship has not yet been established, warranting further investigation.

Therefore, future research will likely focus on the following areas. First, mechanobiology integrated with multi-omics approaches will be leveraged to delineate key differences across anatomical sites and disease stages. Second, a new biomarker and classification system centered on the “mechanics–inflammation–fibrosis loop” will be developed to inform clinical intervention. Third, pivotal molecules such as YAP/TAZ, integrin–FAK signaling, and mechanosensitive ion channels will be explored as potential therapeutic targets. Finally, mechanism-driven, multimodal clinical trials will be designed to validate the efficacy of different therapeutic strategies.

At the clinical level, the therapeutic concept for keloids should also gradually shift from one-time intervention toward the long-term management of a chronic disease, with emphasis on regular follow-up, stage-based intervention, and individualized treatment. In surgical management, greater attention should be paid to measures that modulate the mechanical environment, including incision design based on skin tension lines, tension-reducing suturing, and flap redistribution. Postoperatively, modalities such as NPWT, silicone-based products, and pressure therapy should be rationally combined according to the specific clinical context. As understanding of the interplay between mechanical forces and inflammatory responses continues to deepen, the diagnosis and treatment of keloids are expected to further evolve toward mechanism-oriented, individualized, and multimodal combination strategies, thereby helping patients overcome the current challenges of refractoriness and high recurrence, and opening new directions for clinical management.
